# Glucose Oxidase-Instructed Traceable Self-Oxygenation/Hyperthermia Dually Enhanced Cancer Starvation Therapy

**DOI:** 10.7150/thno.40439

**Published:** 2020-01-01

**Authors:** Ting He, Han Xu, Yifan Zhang, Shijian Yi, Run Cui, Shaojun Xing, Chaoliang Wei, Jing Lin, Peng Huang

**Affiliations:** 1 Marshall Laboratory of Biomedical Engineering, International Cancer Center, Laboratory of Evolutionary Theranostics (LET), School of Biomedical Engineering, Shenzhen University Health Science Center, Shenzhen 518060, China.; 2 Key Laboratory of Optoelectronic Devices and Systems of Ministry of Education and Guangdong Province, College of Optoelectronic Engineering, Shenzhen University, Shenzhen 518060, China.; 3 Department of General Surgery, Shenzhen University General Hospital, Shenzhen 518055, China.

**Keywords:** manganese oxidase, melanin, photothermal therapy, starvation therapy, synergistic therapy.

## Abstract

Cancer theranostics based on glucose oxidase (GOx)-induced starvation therapy has got more and more attention in cancer management. Herein, GOx armed manganese dioxide nanosheets (denoted as MNS-GOx) were developed as cancer nanotheranostic agent for magnetic resonance (MR)/photoacoustic (PA) dual-modal imaging guided self-oxygenation/hyperthermia dually enhanced starvation cancer therapy. The manganese dioxide nanomaterials with different morphologies (such as nanoflowers, nanosheets and nanowires) were synthesized by a biomimetic approach using melanin as a biotemplate. Afterwards, the manganese dioxide nanosheets (MNS) with two sides and large surface area were selected as the vehicle to carry and deliver GOx. The as-prepared MNS-GOx can perform the circular reaction of glucose oxidation and H_2_O_2_ decomposition for enhanced starvation therapy. Moreover, the catalytic activity of GOx could be further improved by the hyperthermia of MNS-GOx upon near-infrared laser irradiation. Most intriguingly, MNS-GOx could achieve “turn-on” MR imaging and “turn-off” PA imaging simultaneously. The theranostic capability of MNS-GOx was evaluated on A375 tumor-bearing mice with all tumor elimination. Our findings integrated molecular imaging and starvation-based synergistic cancer therapy, which provided a new platform for cancer nanotheranostics.

## Introduction

Glucose is an important source of energy in living organisms, the occurrence and development of many diseases are closely related to the glucose metabolism^1^-^2^. Utilization of glucose oxidation through the catalytic reaction of glucose oxidase (GOx) can cut off energy supply to inhibit the tumor growth^3^-^5^, which are known as GOx-based starvation therapy that has attracted more and more attention in cancer management in recent years^4^, ^6^-^11^. However, the starvation therapies based on GOx are restricted by the hypoxic state of tumor tissues, because glucose oxidation reactions need a large amount of oxygen (O_2_)^12^-^13^. Therefore, how to improve the O_2_ level of tumor tissues will directly decide the therapeutic effect of GOx-based starvation therapy.

The products of glucose oxidation are including gluconic acid and hydrogen peroxide (H_2_O_2_). Meanwhile, tumor microenvironments also contain a high content of H_2_O_2_. As we know, the decomposition of H_2_O_2_ can produce water and O_2_, thus promising the melioration of tumor hypoxia. Manganese oxide (MnO_2_) nanomaterials can catalyze decomposition reaction to produce O_2_ to modulate solid tumor hypoxia for effective starvation therapy^14^-^18^. Therefore, the combination of MnO_2_ and GOx will accelerate glucose consumption to improve the starvation therapeutic effect. Recently, MnO_2_-based theranostic agents have been widely explored^14^,^19^-^23^, such as urchin-shaped MnO_2_ nanoparticles^17^, ^24^-^25^, hollow MnO_2_ nanoplatform^26^-^27^, MnO_2_ nanosheets^28^-^31^ and Mn-based layered double hydroxide^32^ and so on. However, most of MnO_2_ nanomaterials need polyethylene glycol (PEG) modification to improve their biocompatibility and water solubility^28^. Melanin, an endogenous biomolecule, has been employed as coating materials to endow nanoplatforms with good biocompatibility, dispersibility, and strong near-infrared (NIR) absorption^33^-^37^. Moreover, melanin-based nanoplatforms have great potential in the biomedical applications of photoacoustic imaging (PAI) and photothermal therapy (PTT).

**Scheme 1:**
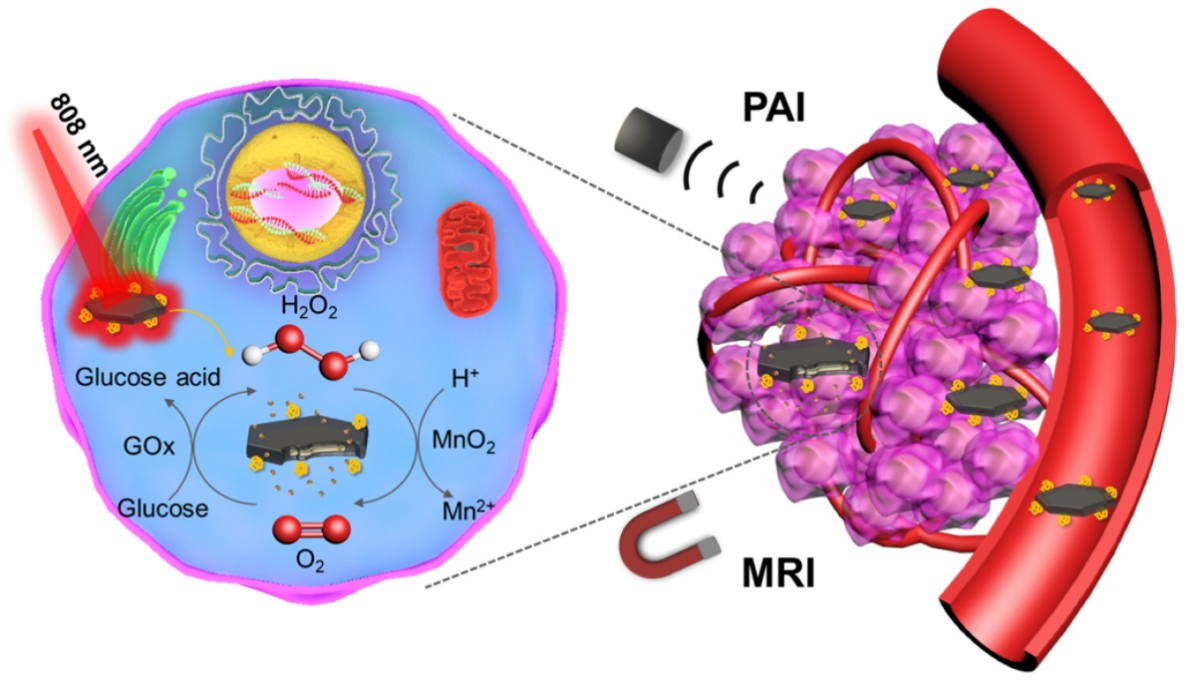
Schematic illustration of MNS-GOx for magnetic resonance (MR)/photoacoustic (PA) dual-modal imaging guided self-oxygenation/hyperthermia dually enhanced starvation cancer therapy.

Herein, we used melanin as the biotemplate to directly synthesize MnO_2_ nanomaterials with different morphologies (such as nanoflowers, nanosheets and nanowires). The melanin coated MnO_2_ have good biocompatibility and strong NIR absorption. Afterwards, GOx armed manganese dioxide nanosheets (denoted as MNS-GOx) were explored for cancer nanotheranostics with following characteristics (Scheme 1): (i) the circular reaction of glucose oxidation and H_2_O_2_ decomposition; (ii) the enhancement of catalytic activity of GOx by the hyperthermia of MNS-GOx; (iii) activatable magnetic resonance (MR)/photoacoustic (PA) dual-modal imaging. The theranostic capability of MNS-GOx was carefully investigated both *in vitro* and *in vivo*. The as-prepared MNS-GOx has great potential in traceable cancer therapy, especially for MR/PA dual-modal imaging guided self-oxygenation/hyperthermia dually enhanced starvation cancer therapy.

## Experimental Section

### Synthesis of MNS

MnO_2_ nanosheets (MNS) were synthesized by the biomimetic synthesis method using melanin as the biomimetic template. Typically, 0.4 mL melanin (2 mg/mL) and 3.2 mL ethanol were added into 48 mL deionized (DI) water. The pH value of the mixed solution was adjusted to 7.4~7.8. Then the solution was heated to 90 °C, 0.32 mL potassium permanganate (KMnO_4_) (0.05 M) was slowly added under vigorous stirring. The system was kept stirring for 30 min at 90 °C. The product was purified and concentrated by an ultrafilter (30 kDa).

### Synthesis of MNS-GOx

GOx was conjugated on the surface of MNS through the 1-(3-Dimethylaminopropyl)-3-ethylcarbodiimide hydrochloride (EDC)/N-Hydroxysuccinimide (NHS) reaction between carboxyl groups of MNS and amine groups of GOx. In brief, 10 mg EDC and 5 mg NHS were added into 4 mL MNS (1 mg/mL). After 2 h, 40 µL GOx (2 mg/mL) was added and the mixture solution was stirred for another 8 h. The MNS-GOx was washed with DI water for several times and suspended in water for further use. In further experiments, the ratios of 200:1 and 1:1 of MNS: GOx were prepared for* in vitro* and* in vivo* experiments, respectively.

### Characterization

The morphologies of MnO_2_ nanomaterials were observed by high-resolution field emission transmission electron microscope (TEM) (JEM-3200FS, JEOL, Japan). The thickness of MNS was measured by atom force microscope (AFM) (MultiMode 8, Bruker, Germany). X-ray diffraction (XRD) pattern of MNS was detected by X-ray diffractometer (D8 Advance, Bruker, Germany). Dynamic light scattering (DLS) and Zeta potential was measured on Zetasizer Nano-ZS90 (Malven, England). Fourier-transform infrared (FT-IR) spectra were collected on a FT-IR spectrometer (Spectrum Two, PerkinElmer, USA). UV/Vis absorption spectra were measured on a Cary 60 UV/Vis spectrophotometer (Agilent Technologies, Santa Clara, CA, USA). The Mn element concentration of MNS was quantified by an inductively coupled plasma-atomic emission spectrometry (ICP-AES) (JY 2000-2, Horiba, France).

### pH-Responsive Degradations of MNS and MNS-GOx

The pH-responsive degradations of MNS and MNS-GOx were monitored by UV/Vis spectrophotometer. The optical density (OD) at 400 nm of MNS (100 µg/mL) or MNS-GOx (100 µg/mL) in PBS solutions (5 mM glucose, pH 7.4) was measured over time. Additionally, 4 mL of MNS (100 µg/mL) or MNS-GOx (100 µg/mL) was loaded into a dialysis bag with the molecular weight cut-off of 3500 Da. Then the bag was immersed into 50 mL of buffer solutions with different pH values (5 mM glucose pH 5.0, 6.0, 7.4) under vigorous stirring at room temperature. At different time points, 2 mL of buffer solution was taken out and the released Mn^2+^ was detected by ICP-AES. Meanwhile, an isometric buffer solution was added to keep the total volume same.

### Photothermal Performance of MNS

The aqueous solutions of MNS (0~200 µg/mL) were stored in Eppendorf tubes, and exposed to 808 nm laser at power density of 1 W/cm^2^ for 3 min. The photothermal stability of MNS (200 µg/mL) were irradiated upon different laser power density of 1 W/cm^2^ for four on/off cycles (on: 2 min, off: 6 min). Real-time thermal imaging was recorded by a SC300 infrared camera (FLIR, Arlington, VA) and quantified by FLIR Examiner software.

### *T*_1_ Relaxivity of MNS and MNS-GOx

MNS and MNS-GOx with different Mn element concentration (0.2, 0.4, 0.6, 0.8, 1.0 mM) were dispersed in PBS solutions with different pH values (5, 6, 7.4) for 4 h. Then the *T*_1_ relaxivity was detected by a 3 T clinical MRI scanner (UMR 790, United-Imaging, Shanghai, China). An fast spin echo (FSE) sequence with 19 different values of T_1_ (TR (repetition time)=5000 ms; TE (ehco time) =100, 150, 200, 250, 300, 350, 400, 450, 500, 550, 600, 700, 800, 900, 1000, 1200, 1300, 1500, 2000 ms) for T_1_ measurements. T_1_ relaxation times were calculated by fitting the signal intensities with increasing T_1_ to I_(t)_=I_0_[1-k•exp(-t/T_1_)] by using a nonlinear least-squares fit of the Levenberg-Marquardt algorithm.

### Catalytic Activity of GOx

30 µL of MNS-GOx (1 mg/mL) or GOx (1 mg/mL) was added into 1 mL of glucose solution (30 mM) under oxygen gas flow (5 mL/min). The glucose concentrations were immediately detected by Yuwell 590 glucometer at different time points (0, 2, 4, 6, 8 min). Moreover, the glucose solutions were heated up to 30, 40, 50 or 60 ºC. The kinetic parameters (V_max_) of GOx obtained from the Michaelis-Menten plots at different temperatures^38^-^41^. Meanwhile, the catalytic activity of GOx in MNS-GOx was investigated before and after laser irradiation (808 nm laser, 1 W/cm^2^ for 5 min).

### *In Vitro* Synergistic Therapy

For *in vitro* synergistic therapy, four parallel groups were set: control, MNS + laser, MNS-GOx, and MNS-GOx + laser. A375 cells were seeded into a 96-well plate at 10^4^/well and then cultured at 37 °C for 24 h. The cells were incubated with MNS or MNS-GOx (0~1 mM) for 4 h and then exposed to an 808 nm laser at 1 W/cm^2^ for 5 min. Then the old dulbecco's modified eagle medium (DMEM) media were removed and 5 mg/mL 3-(4,5-dimethylthiazol-2-yl)-2,5-diphenyltetrazolium bromide (MTT) in 100 μL DMEM media was added into each well. After co-incubation for another 4 h, the old DMEM media were replaced with 150 μL dimethylsulfoxide (DMSO) per well, and the absorbance at 490 nm was monitored by a microplate reader. The cytotoxicity was finally expressed as the viabilities of different-treated cells in contrast to the untreated control cells.

A375 cells were seeded into a 12-well plate at 7*10^4^/well and then cultured at 37 °C for 24 h. MNS or MNS-GOx (1 mM, DMEM media) was added into each well and co-incubated for 4 h. Then the cells were exposed to an 808 nm laser at 1W/cm^2^ for 5 min. After co-incubation for another 24 h, the cells were co-stained by Calcein acetoxymethyl ester (calcein AM) and propidium iodide (PI), and then imaged using an Olympus FV1000 fluorescent microscope.

### *In Vivo* MR/PA Dual-Modal Imaging

The tumor-bearing nude mice (∼20 g) were intratumoral injected with MNS or MNS-GOx (5 mg/kg). Magnetic resonance imaging (MRI) was performed by a UMR 790 3.0T (United-Imaging, Shanghai, China). T_1_-weighted images were acquired by FSE sequence at 0, 0.5, 1, 4, 8, 24 h and the parameters were as follows: TR=700 ms; TE=14.3 ms; Flip Angle=145 º; matrix size, 160 × 160; slice thickness, 1.5 mm. Signal intensities were measured in defined regions of interest (ROI) with software named Image J. The PA images were recorded by a Vevo LAZR2100 system (VisualSonics Inc. New York, NY) equipped with a 40 MHz, 256-element linear array transducer on tumors.

### *In Vivo* Synergistic Therapy

A375 cells were implanted subcutaneously into nude mice (∼20 g). *In vivo* treatment was performed when the tumor reached 6 mm in average diameter (10 days after implant). The mice were divided into six groups: PBS as the control group; PBS with laser irradiation (PBS + laser); MNS; MNS-GOx; MNS with laser irradiation (MNS + laser); MNS-GOx with laser irradiation (MNS-GOx + laser). For laser irradiation groups, 808 nm laser with the power of 0.6 W/cm^2^ was used to irradiate tumor tissues for 5 min after intratumoral injection (dose: 5 mg/kg). For each group, the tumor volumes and body weight were measured every two days, and volume of tumors was calculated as (tumor length) × (tumor width/2)^2^. Relative tumor volume was calculated as V/V_0_ (V_0_ is the tumor volume when the treatment was initiated).

### Hemolysis Assay

The red blood cells (RBCs) were isolated from serum by centrifugation of the mixture containing 0.5 mL blood sample and 1 mL PBS solution at 4500 rpm for 3.5 min, then washed the RBCs over five times and diluted the purified cells to 5 mL. Then 0.3 mL volume diluted RBCs suspension was added to quadruple volume of PBS solution with different concentrations of MNS (12.5 to 400 µg/mL). The mixtures were vortexed and kept to stand for 4 h at room temperature. Samples were then centrifuged to measure the absorbance of the supernatants at 541 nm by an UV-vis spectroscopy. RBCs treated with deionized water and PBS were set as positive and negative controls.

### *Ex vivo* Histological Staining

Tumor tissues were collected from tumor-bearing mice in different groups at day 16 and sectioned into slices for hematoxylin and eosin (H&E) staining. For the toxicity evaluation of MNS or MNS-GOx, mice with intratumoral injection of MNS or MNS-GOx (dose: 5 mg/kg) were sacrificed at day 30 (3 mice per group). Then tumor tissues and major organs (including heart, liver, spleen, lung, kidney and tumor.) were collected, fixed in 10% neutral buffered formalin, embedded in paraffin, cut into 4 µm sections and stained with H&E. Finally, the images of these histological tissue sections were obtained by a BX41 bright field microscopy (Olympus).

### Statistical Analysis

Data were presented as mean ± standard deviation (SD). Statistical differences among experimental groups were analyzed using one-way analysis of variance (ANOVA) followed by two-tailed Student's t test. *P* < 0.05 was considered as statistically significant.

## Results and Discussions

### Preparation and Characterization of MNS and MNS-GOx

**Figure 1:**
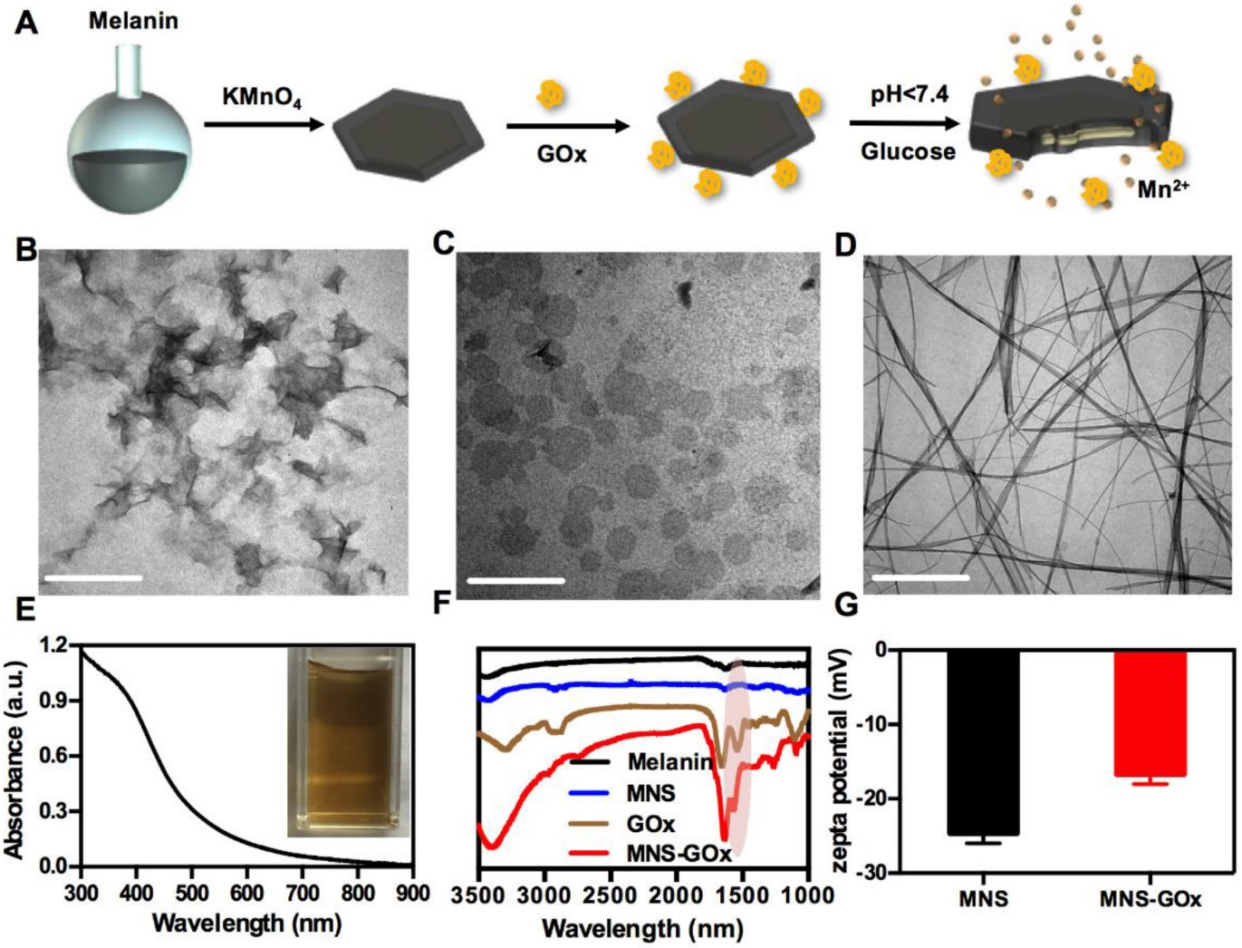
(A) Scheme of the synthesis process of MNS-GOx. TEM images of nanoflowers (B), nanosheets (C), nanowires (D). Scale bar: 200 nm. (E) UV-vis-NIR absorption spectrum of MNS. (F) FT-IR spectra of melanin, MNS, GOx and MNS-GOx. (G) Zeta potentials of MNS and MNS-GOx.

MNS were prepared by one-step reduction of KMnO_4_ using melanin as a template under neutral conditions, and then modified with GOx by a cross-linker, as shown in **Figure 1A**. Due to an abundant of catechol groups of melanin, it can anchor on the surface of metals oxides^42^. Therefore, the morphologies of MnO_2_ were strongly depended on the solubility of melanin under different reaction conditions. The as-prepared MnO_2_ nanomaterials were nanoflowers **(Figure 1B)** at acidic condition (pH < 7), ultrathin nanosheets **(Figure 1C)** at neutral condition (pH 7) and nanowires **(Figure 1D)** at alkaline condition (pH 10). The nanoflowers can be formed when the decomposition rate of KMnO_4_ was faster in acidic condition and the nanowires were probably formed when the solubility of melanin increased in alkaline solution^43^. The MNS with two sides and large surface area was chosen as the vehicle to carry and deliver GOx. As shown in **Figure S1**, the hydrodynamic diameter of MNS was about ~70 nm measured by DLS. High resolution TEM image of MNS indicated that the lattice fringe spacing of MNS were 0.232 and 0.288 nm **(Figure S2)**, which can be attributed to (311) and (411) according to the XRD pattern **(Figure S3)**. EDS spectrum confirmed the high concentration of manganese and oxygen elements in MNS, which is further supported by element mapping images in **Figure S4**. As shown in **Figure S5**, the thickness of MNS was ~ 2 nm, which indicated the MNS with ultrathin sheet structure.

Melanin as the biotemplate plays an important role in regulating the morphology of MnO_2_ nanomaterials during the synthesis process. It can be adsorbed on the surface of MNS through the abundant catechol groups of melanin^42^, ^44^. As shown in **Figure 1E**, UV-vis-NIR absorption spectrum of MNS contained the broad absorbance of melanin^35^ and the characteristic peak of MnO_2_ (~400 nm)^44^. The absorbance at 808 nm of MNS was positively correlated with MnO_2_ concentration, and the typical equation was Y = 0.003X + 0.0102 (R^2^ = 0.9736) **(Figure S6)**. The photothermal effect of MNS exhibited a MNS concentration-dependent under 808 nm laser irradiation **(Figure S7A-C)**. After 4 cycles of laser irradiation, MNS still remained its excellent photothermal conversion property, suggested that MNS has a great photostability **(Figure S7D)**. The melanin modified MnO_2_ had a large number of carboxyls for further surface modification. GOx can be covalently conjugated onto the surface of MNS by EDC/NHS reaction. FT-IR spectrum of MNS-GOx showed a new peak at about 1650 cm^-1^, indicating that GOx was successfully grafted onto the surface of MNS **(Figure 1F)**. The zeta potentials of MNS and MNS-GOx were -24.6 and -16.7 mV, respectively **(Figure 1G)**. These results indicated GOx was successfully loaded by MNS.

### pH-Responsive Degradation of MNS-GOx

**Figure 2:**
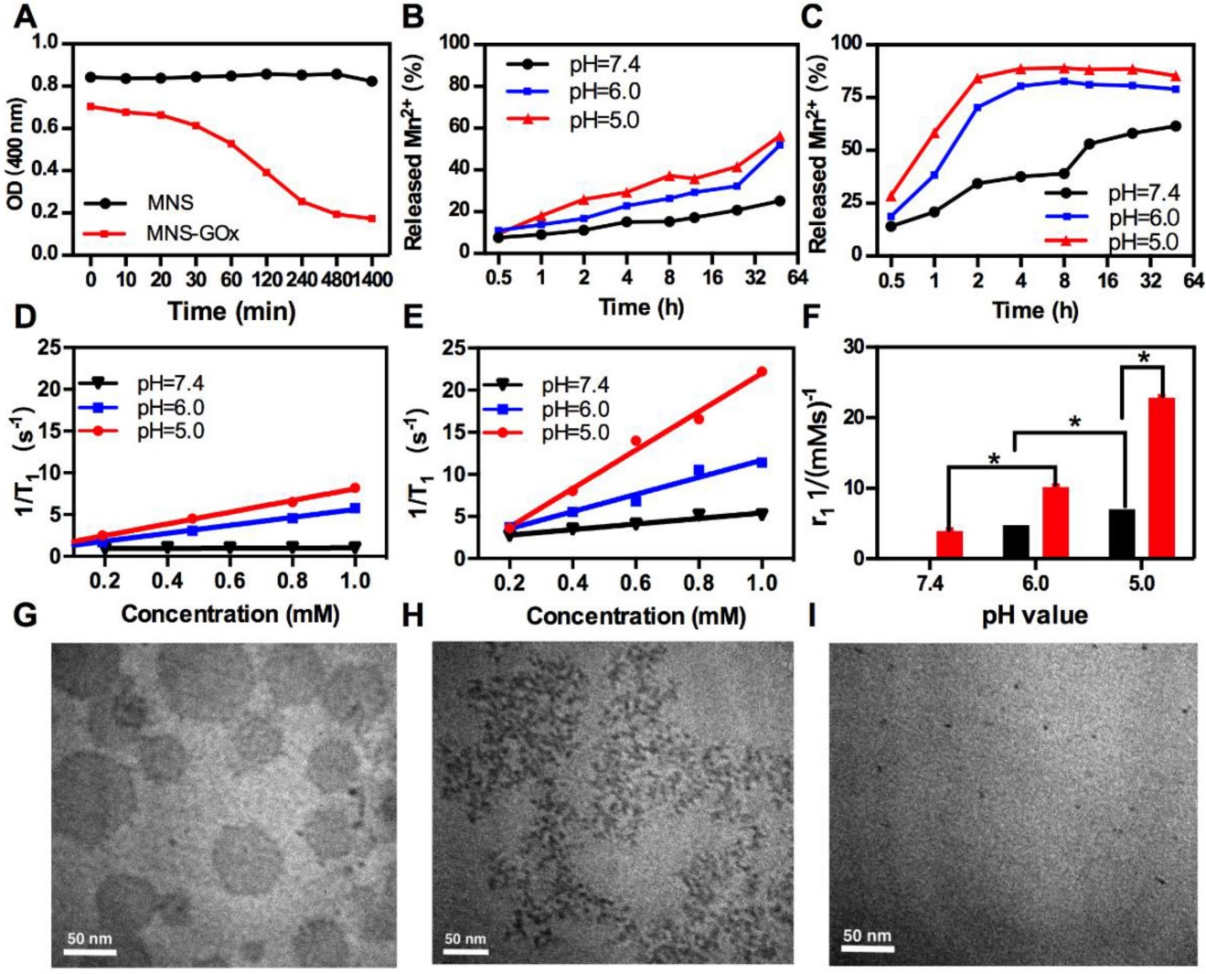
(A) The OD at 400 nm of MNS and MNS-GOx in PBS solutions (5 mM glucose, pH 7.4). The quantified concentration of Mn^2+^ released from MNS (B) or MNS-GOx (C) in PBS solutions (5 mM glucose, pH 5.0, 6.0, 7.4). The profile of 1/T_1_ vs Mn element concentration in MNS (D) and MNS-GOx (E) after 4 h incubation in PBS solutions (5 mM glucose, pH 5.0, 6.0, 7.4). (F) The corresponding T_1_ relaxivity of MNS and MNS-GOx solutions. TEM images of MNS-GOx in PBS solutions (5 mM glucose, pH 7.4), before (G), 17 min (H), and 30 min (I).

MnO_2_ nanomaterials can be decomposed into Mn^2+^ in the tumor microenvironment for MRI and alleviating the hypoxia of tumor^24^, ^28^, ^45^-^46^. As shown in **Figure 2A**, the OD at 400 nm of MNS-GOx decreased from 0.70 to 0.17 within 8 h in 5 mM glucose solution, while that of MNS kept stable at about 0.84. Because MNS-GOx can catalyze glucose to produce gluconic acid and H_2_O_2,_ which promoted the degradation of MNS. The color of MNS-GOx solution was changed from brown to colorless over time **(Figure S8)**. Moreover, the quantified concentration of Mn^2+^ released from MNS or MNS-GOx was further verified the degradation of MNS. As shown in **Figure 2B-C**, the Mn^2+^ released from MNS-GOx can reach 89% at pH 5, but it was only 56.3% of MNS at the same condition. The concentration of released Mn^2+^ will directly affect the following longitudinal relaxivity (r_1_). Because the released Mn^2+^ had five unpaired electrons, Mn-based contrast agents have been widely explored as T_1_ contrast agents. Based on the profile of 1/T_1_ vs Mn element concentration of MNS **(Figure 2D)** and MNS-GOx **(Figure 2E)**, the r_1 MNS_ increased from 0.084 to 7.0 mM^-1^s^-1^ from pH 7.4 to 5.0, while r_1 MNS-GOx_ increased from 3.3 to 22.8 mM^-1^s^-1^**(Figure 2F)**. The r_1 MNS_ enhancement is due to the pH-responsive degradation of MNS, while the r_1 MNS-GOx_ enhancement is due to pH/glucose dual-responsive degradation of MNS, which accelerated the release of Mn^2+^. In order to observe the degradation process of MNS-GOx, their morphology change was recorded by TEM imaging. As shown in **Figure 2G-I**, when MNS-GOx was incubated with 5 mM glucose, most of MNS was decomposed at 17 min, and no MNS can be found at 30 min. These results suggested MNS-GOx exhibited pH/glucose dual-responsive performance.

### *In vitro* Synergistic Therapy

**Figure 3:**
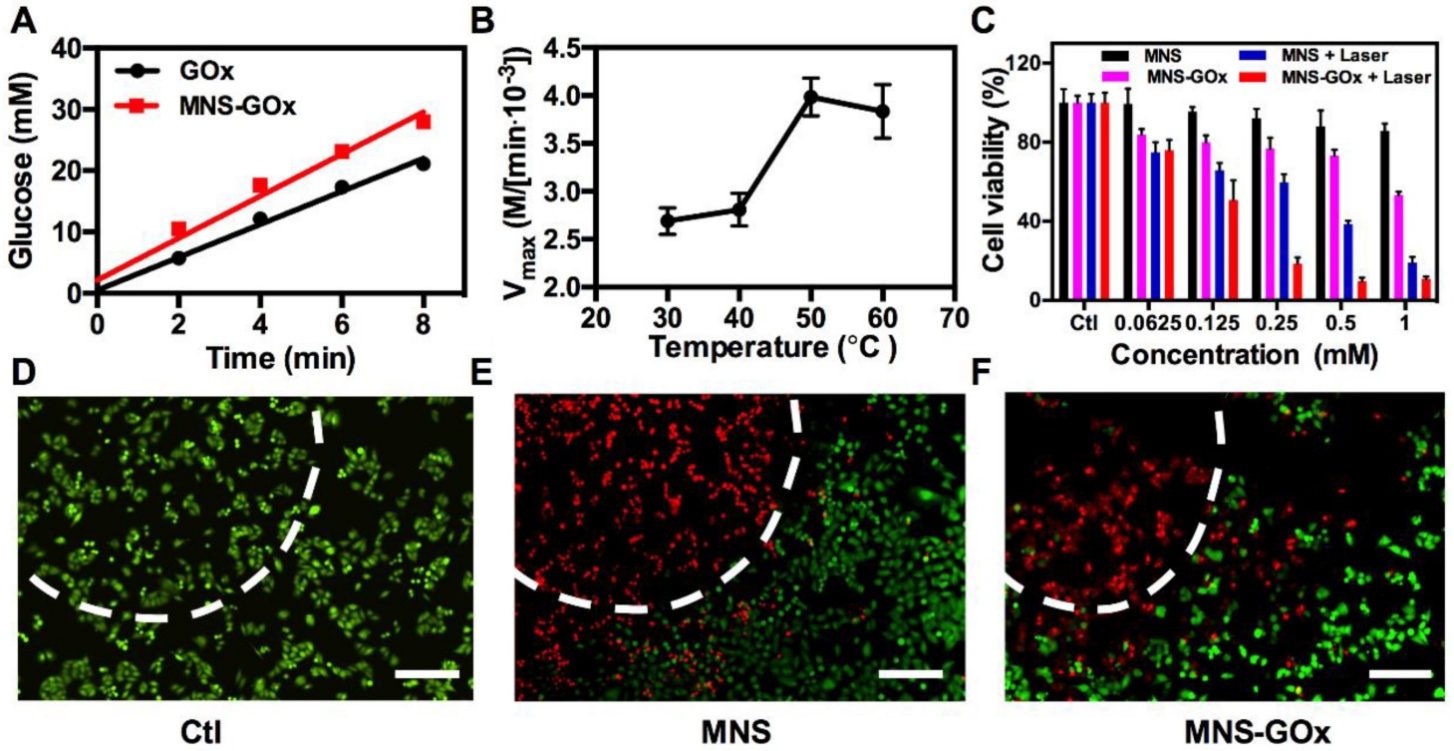
(A) The glucose oxidation rates of GOx and MNS-GOx at room temperature. (B) Kinetic parameters (V_max_) of GOx obtained from the Michaelis-Menten plots at different temperatures. (C) A375 cell viability with four different treatments, including: control, MNS, MNS-GOx, MNS + laser, MNS-GOx + laser. Fluorescence images of Calcein AM (live cells, green fluorescence) and propidium iodide (PI) (dead cells, red fluorescence) co-stained A375 cells with different treatments, including control (D), MNS (E), MNS-GOx (F) (100 μg mL^-1^) for 12 h with/without laser irradiation (808 nm, 1 W cm^-2^, 5 min). Scale bar 100 µm.

MnO_2_ nanomaterials can catalyze the decomposition reaction of H_2_O_2_ to produce O_2_, which can promote the reaction of glucose oxidation^16^. As shown in **Figure 3A**, the glucose oxidation rate of GOx and MNS-GOx were 2.69 and 3.43 mM/min, respectively. The catalytic activity enhancement of GOx by MNS can be attributed to the self-oxygenation of MNS-GOx during the glucose oxidation reaction that is an O_2_-depandent reaction. Afterwards, we investigated the catalytic activity of GOx at different temperature. The glucose concentrations were recorded by a glucometer, and extra O_2_ was supplied into glucose solution for sufficient O_2_ during the reaction. The results shown that V_max_ of MNS-GOx increased from 2.69✕10^3^ M/min at 30 °C to 3.43✕10^3^ M/min at 50 °C **(Figure 3B)**. It was consistent with previous reports that the maximum activity of GOx at 50 °C 40-41, 47-48. Encouraged by the catalytic activity enhancement of GOx at high temperature, *in vitro* experiments were conducted on A375 cells for hyperthermia-enhanced synergistic therapy. MNS or MNS-GOx was incubated with A375 cells for 24 h, and then irradiation with or without 808 nm laser (1 W/cm^2^, 5 min). As shown in** Figure 3C**, cells incubated with MNS kept their viability over 81%. Cell viabilities of MNS + laser, MNS-GOx and MNS-GOx + Laser groups showed concentration-dependent therapeutic effect. Importantly, MNS-GOx + laser group exhibited better therapeutic effect than that of MNS-GOx only. The synergistic therapeutic effect was further evidenced by live/dead cell staining **(Figure 3D-F)**. No dead cells were found in control group **(Figure 3D)**. For MNS + laser and MNS-GOx + laser groups, most of cells were dead in laser spots **(Figure 3E-F)**. These results indicated the catalytic activity of GOx could be further improved by the hyperthermia of MNS-GOx upon 808 nm laser irradiation. Additionally, we assessed the catalytic activity of GOx before and after laser irradiation. As shown in **Figure S9**, the glucose reaction rates of MNS-GOx kept similar, which suggested the laser irradiation (808 nm, 1 W/cm^2^, 5 min) can preserve the catalytic activity of GOx.

### *In Vivo* Dual-Modal Imaging

**Figure 4:**
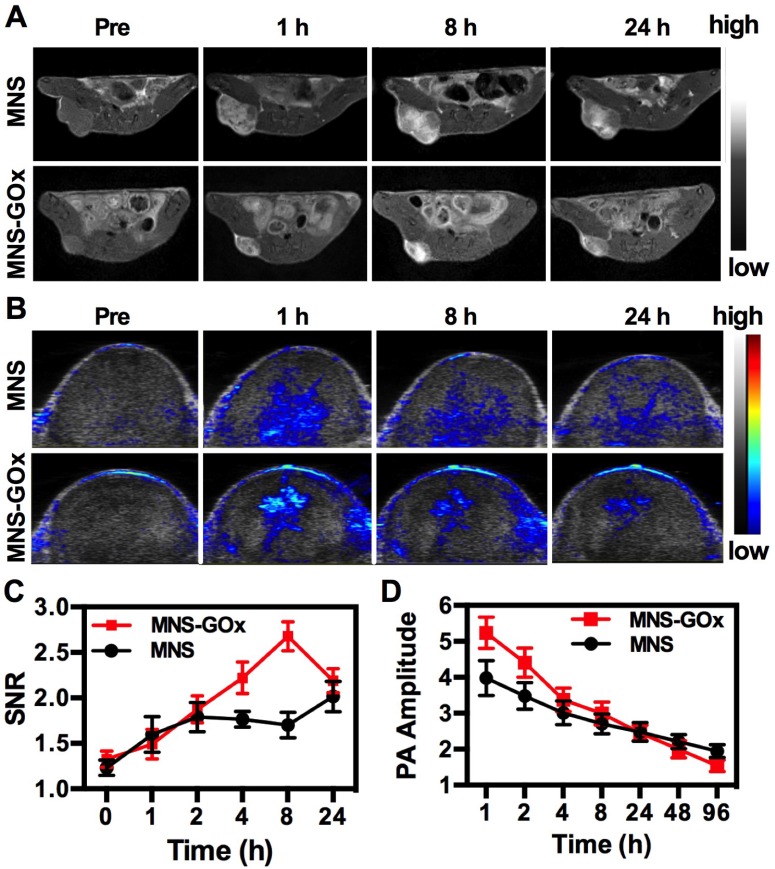
T_1_-weigthed MR (A) and PA images of mice treated with MNS or MNS-GOx at 0, 1, 8, 24 h post-injection. (C) The corresponding MR signal SNR analysis of tumor tissues in (A). (D) The corresponding PA signals of tumor tissues in (B).

MNS-GOx was composed of Mn element and melanin, which can be used as contrast agents for MRI and PAI, respectively. *In vivo* MR/PA dual-modal imaging was performed on the A375 tumor-bearing mice. MNS or MNS-GOx was intratumoral injected into A375 tumor-bearing mice at 5 mg/kg dose. Then the treated mice were scanned on 3T clinic MRI scanner. Both MNS and MNS-GOx displayed an obvious T_1_-weighted enhancement in tumor tissues **(Figure 4A)**. Compared with MNS treated mice, mice in MNS-GOx group exhibited stronger contrast effect at each scan time point. Because MNS-GOx are pH/glucose dual-responsive, it can release more Mn^2+^ in tumor microenvironment than MNS, and the concentration of released Mn^2+^ will directly affect the longitudinal relaxivity and MRI contrast effect. So MNS-GOx group exhibited stronger contrast effect compared with MNS group. On the contrary to the increase of MR signal, PA signal of gradually decreased after injection over time **(Figure 4B, Figure S10)**. As shown in **Figure 4C**, the quantified MR signal/noise ratio (SNR) of tumors treated with MNS-GOx gradually increased to the peak value of 2.67 at 8 h post-injection, while the SNR of tumors treated with MNS was only increased to 2.0 at 24 h post-injection. As shown in **Figure 4D**, the quantified PA signals of tumors treated with MNS or MNS-GOx gradually decreased over time. These results suggested MNS-GOx could achieve “turn-on” MR imaging and “turn-off” PA imaging simultaneously. MRI can offer high resolution contrast for soft tissue without tissue depth limits, but restricted by its poor sensitivity and time-consuming. PAI is convenient and time-saving possesses high optical imaging contrast, but limited imaging depth. So the dual-model imaging can integrate different information to provide accurate diagnosis. A “turn on” MRI can light the deep tissues for precision cancer diagnosis, and the “turn off” PAI can monitor the treatment progress for individual treatment.

### *In Vivo* Synergistic Therapy

**Figure 5:**
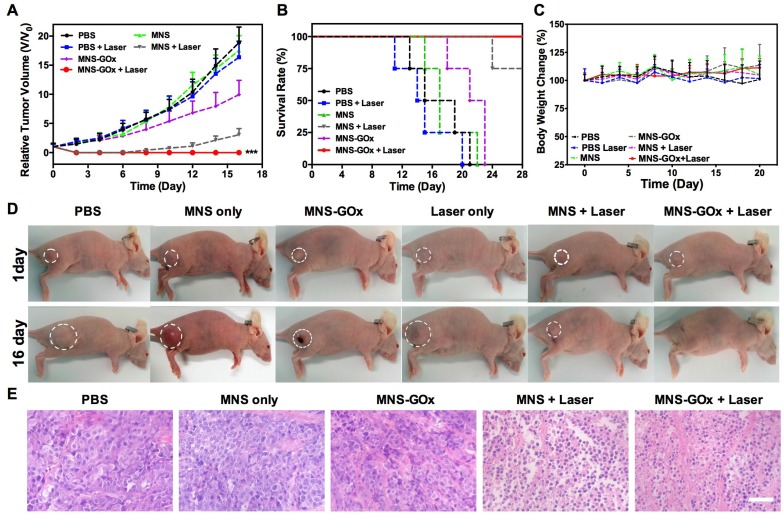
(A) Tumor growth curve, (B) survival curve and (C) body weight of A375 tumor-bearing mice (PBS, laser only, MNS, MNS+laser, MNS-GOx and MNS-GOx+laser) during the treatment process. (D) Digital photographs of A375 tumor-bearing mice of six groups after treatments. (E) H&E staining images of tumor sections harvested from A375 tumor-bearing mice (PBS, MNS only, MNS-GOx, MNS+laser and MNS-GOx+laser) after treatments. Scale bar: 100 µm. ***p<0.001.

Based on *in vitro* synergistic PTT/starvation therapy effect, the *in vivo* synergistic therapy was conducted on A375 tumor-bearing mice. Mice were randomly divided into six groups: PBS, laser only, MNS, MNS+laser, MNS-GOx and MNS-GOx+laser. For all groups, the intratumoral injection dose was 5 mg/kg. For laser groups, 808 nm laser (0.6 W/cm^2^, 5 min) was immediately irradiated on A375 tumor tissues after injection. The tumor volumes were measured every 2 days during 16 days. As shown in **Figure 5A**, the tumors of PBS and PBS + laser groups grew very fast, while MNS-GOx treated group shown great suppression effect compared with MNS group. Interestingly, the tumors of MNS-GOx + laser group were completely eliminated; while MNS + laser group began to regrow at 10 days after treatment.

On the one hand, the increase of tumor local temperature upon laser irradiation can improve the catalytic activity of GOx, thus consuming more intratumoral glucose(∼50 °C, **Figure S11**), on the other hand, MNS-GOx can perform the circular reaction of glucose oxidation and H_2_O_2_ decomposition for enhanced starvation therapy. The self-oxygenation/hyperthermia dually enhanced starvation cancer therapy shown higher tumor suppression effect than any single treatment. Furthermore, the survival rate of mice in MNS-GOx group was greatly prolonged **(Figure 5B)**. All treated mice had no obvious body weight change during different treatments **(Figure 5C)**. The photographs of A375 tumor-bearing mice further evidenced the synergistic PTT/starvation effect **(Figure 5D)**. Hematoxylin and eosin (H&E) staining images of tumor sections after treatments also indicated an obviously membrane fragmentation or shrinkage of nuclei in MNS + laser group and MNS-GOx + laser group **(Figure 5E)**. But no significant damage or inflammation from H&E stained images of main organs (heart, liver, spleen, lung, kidney), negligible change of blood biochemistry results, and the hemolysis rates of virous concentrations (15.5-400 µg/mL) of MNS were lower than 5.5% **(Figure S12-14)**. These findings indicated that MNS-GOx exhibited a great potential in hyperthermia enhanced starvation synergistic therapy.

## Conclusions

In summary, three different morphologies of MnO_2_ nanomaterials were successfully synthesized by a biomimetic approach using melanin as a biotemplate at different pH conditions. Afterwards, GOx loaded MnO_2_ nanosheets (MNS) was explored as cancer nanotheranostics, especially for self-oxygenation/hyperthermia dually enhanced starvation therapy. The as-prepared MNS can decompose H_2_O_2_ to supply O_2_ for the GOx catalyzed glucose oxidation reaction, thus promising the circular reaction of H_2_O_2_ decomposition and glucose oxidation. Moreover, the catalytic activity of GOx could be further improved by the hyperthermia of MNS-GOx upon NIR laser irradiation. Most intriguingly, MNS-GOx could achieve “turn-on” MR imaging and “turn-off” PA imaging simultaneously. The theranostic capability of MNS-GOx was evaluated both *in vitro* and *in vivo*. Finally, the as-prepared MNS-GOx exhibited pH/glucose dual-responsive performance, activatable MR/PA dual-modal imaging, and hyperthermia enhanced starvation synergistic cancer therapy, which provided a new nanoplatform for cancer nanotheranostics.

## Supplementary Material

Supplementary figures.
