# Supplementary material for: Glucose Oxidase-Instructed Traceable Self-Oxygenation/Hyperthermia Dually Enhanced Cancer Starvation Therapy
Source: Theranostics. 2020 Jan 1;10(4):1544–54. doi: 10.7150/thno.40439 (PMC6993236; doi:10.7150/thno.40439)
Supplement: Supplementary file 1 — Supplementary figures. [file thnov10p1544s1.pdf]

Supporting Information

Glucose Oxidase-Instructed Traceable Self-Oxygenation/Hyperthermia Dually Enhanced Cancer Starvation Therapy

Ting He[a,b‡], Han Xu[a‡], Yifan Zhang,[a] Shijian Yi,[c] Run Cui,[a] Shaojun Xing,[a] Chaoliang Wei,[a] Jing Lin,[a] Peng Huang[a,*]

[a] Marshall Laboratory of Biomedical Engineering, International Cancer Center, Laboratory of Evolutionary Theranostics (LET), School of Biomedical Engineering, Shenzhen University Health Science Center, Shenzhen 518060, China

[b] Key Laboratory of Optoelectronic Devices and Systems of Ministry of Education and Guangdong Province, College of Optoelectronic Engineering, Shenzhen University, Shenzhen 518060, China

[c] Department of General Surgery,Shenzhen University General Hospital, Shenzhen 518055, China

Corresponding author:

Peng Huang

Addresses: 1066 Xueyuan Boulevard Shenzhen University Health Science Center, Shenzhen, 518060, China. E-mail: peng.huang@szu.edu.cn (Peng Huang)

‡ These authors contributed equally to this work.

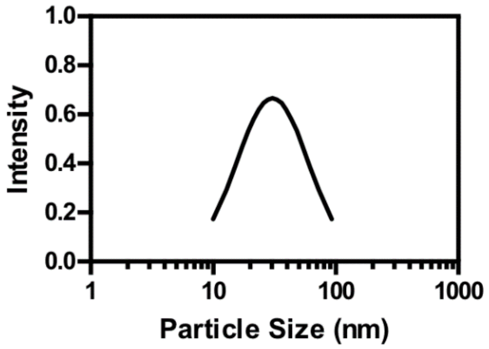

Figure S1 Particle size distribution of MNS.

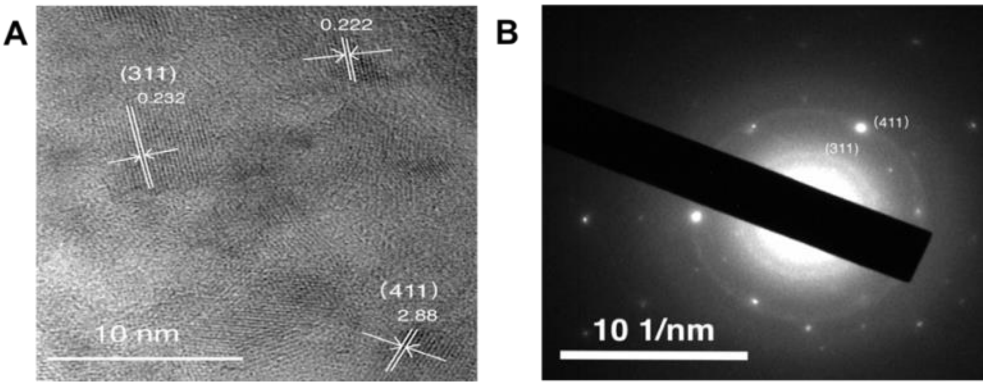

Figure S2 HRTEM images (A) and SAED pattern (B) of MNS .

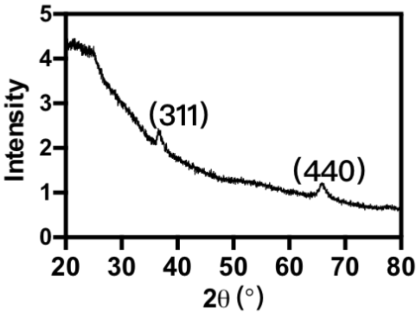

Figure S3 XRD pattern of MNS (JCPDS Card 80-1089).

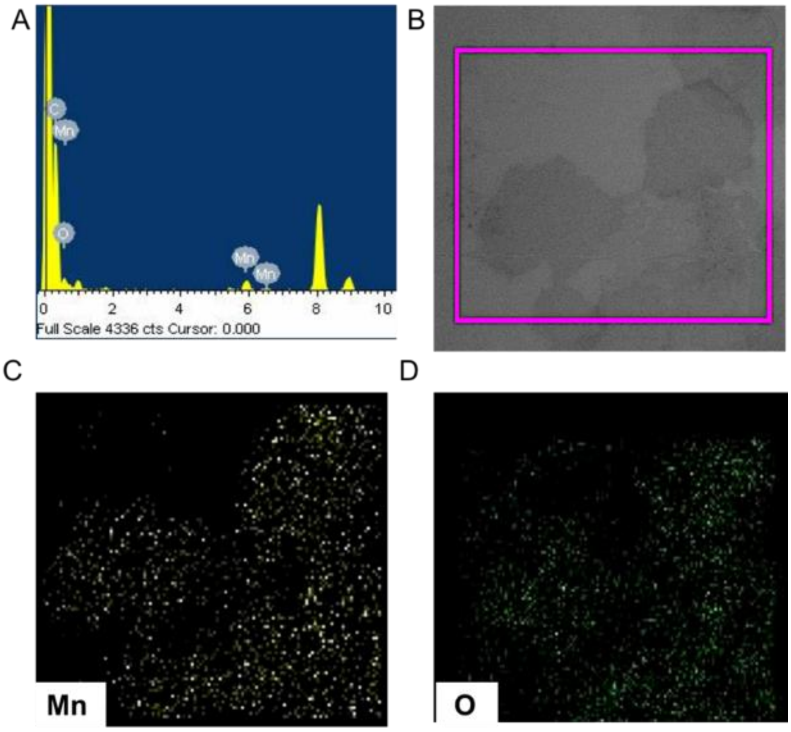

Figure S4 (A) EDX spectra of MNS. EDX elemental mapping of MNS (B) and the corresponding elements: Mn (C), O (D).

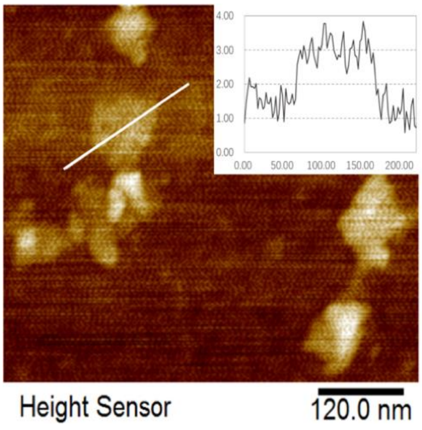

Figure S5 AFM image and its thickness of MNS.

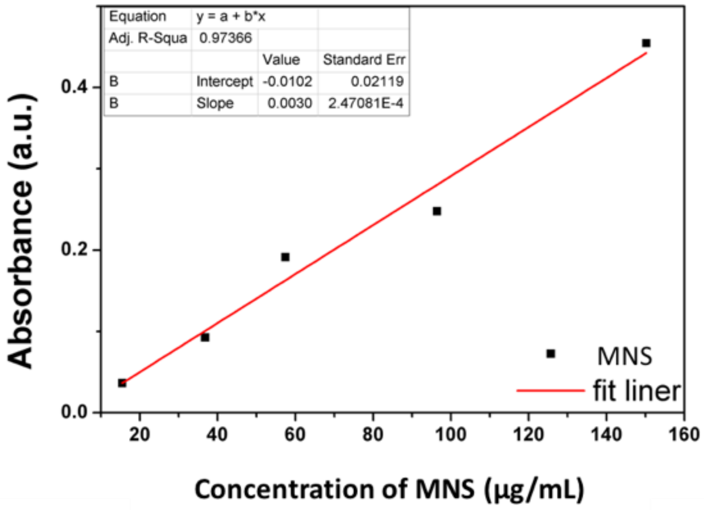

Figure S6 Optical density (OD) of MNS at 808 nm as a function of concentration, described by Y=0.003X-0.0102 (R^2=0.9736)

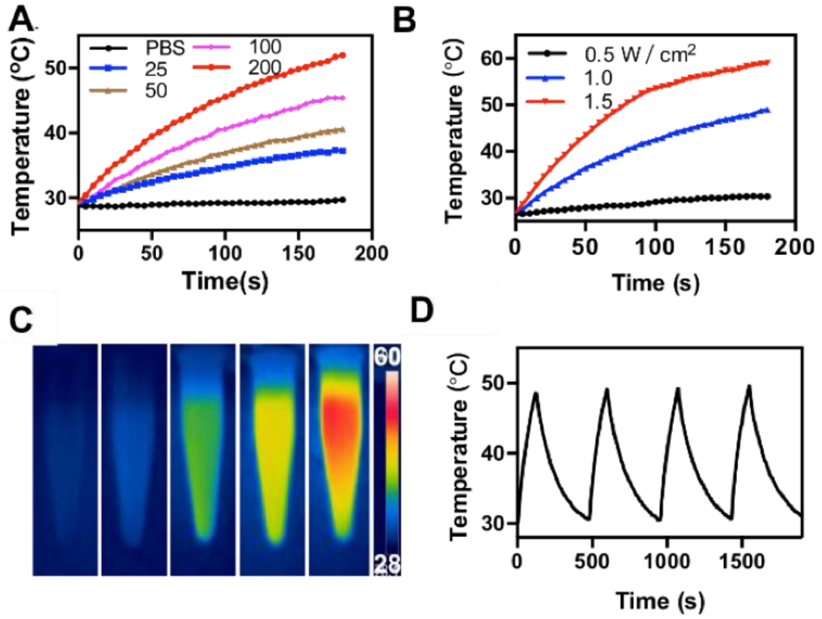

Figure S7 (A) The 808 nm laser-induced heat generation of PBS, 25, 50, 100, 200 μg/mL MNS aqueous solution with laser power density of 1 W/cm^2. (B) The temperature of 200 μg/mL MNS solution irradiated with 0.5, 1, 1.5 W/cm^2 808 nm laser. (C) Thermal images of PBS, 25, 50, 100, 200 μg/mL MNS after 3 min exposed to 1 W/cm^2 808 nm laser. (D) The temperature of 200 μg/mL MNS solution irradiated by an 808 nm laser (1 W/cm^2) for four on/off cycles (on: 2 min, off: 6 min).

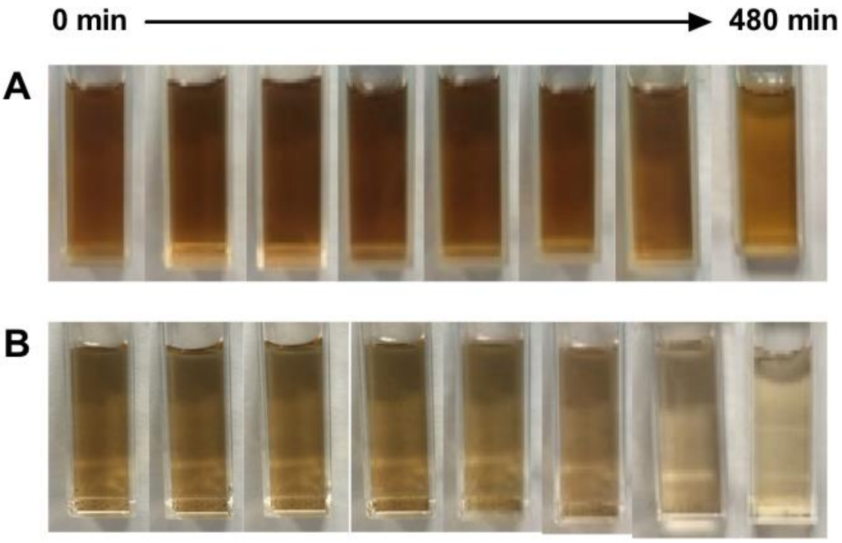

Figure S8 Digital photos of MNS (A) and MNS-GOx (B) in 5 mM glucose PBS pH=7.4

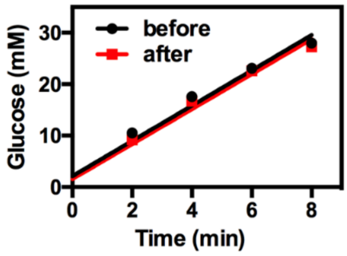

Figure S9 Glucose reaction rate of MNS-GOx before and after 808 nm laser (1 W/cm^2, 5 min) irradiation at 30℃.

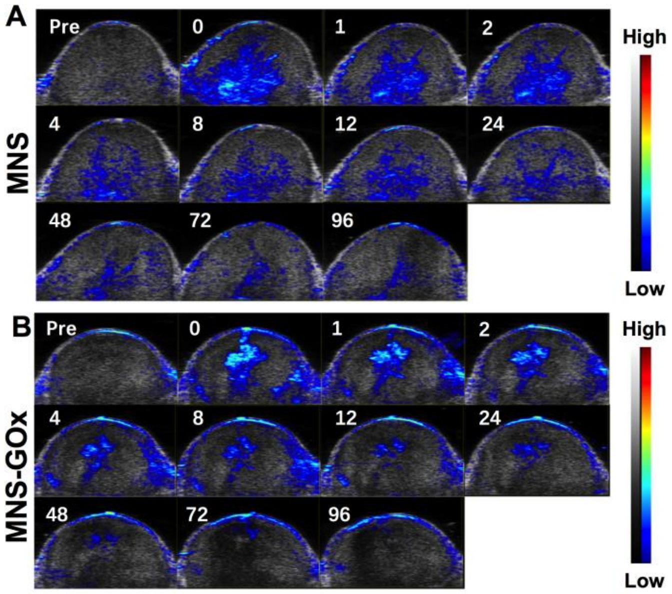

Figure S10 PA images of mice treated with MNS and MNS-GOx, the images were recorded at 0, 1, 2, 4, 8, 12, 24, 48, 72, 96 h.

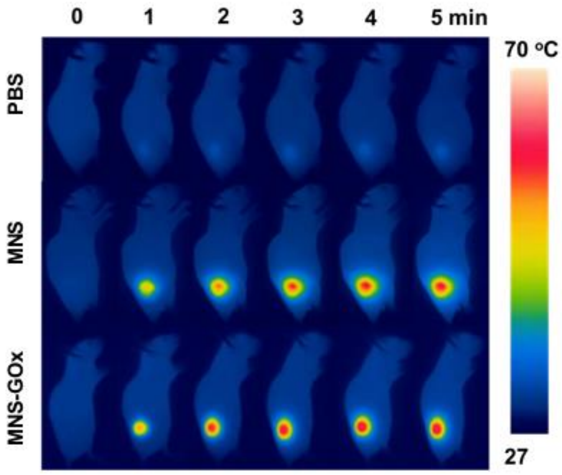

Figure S11 Thermal images of tumor irradiation by an 808 nm laser (1 W/cm^2) after different treatments at a series of time points.

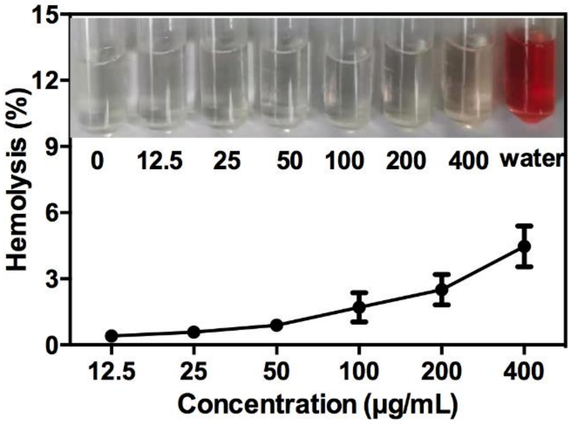

Figure S12 Hemolysis analysis of MNS solution at various concentrations. The mixtures were centrifuged after kept standing for 4 h, then the absorbance of supernatant was measured to detect the hemoglobin.

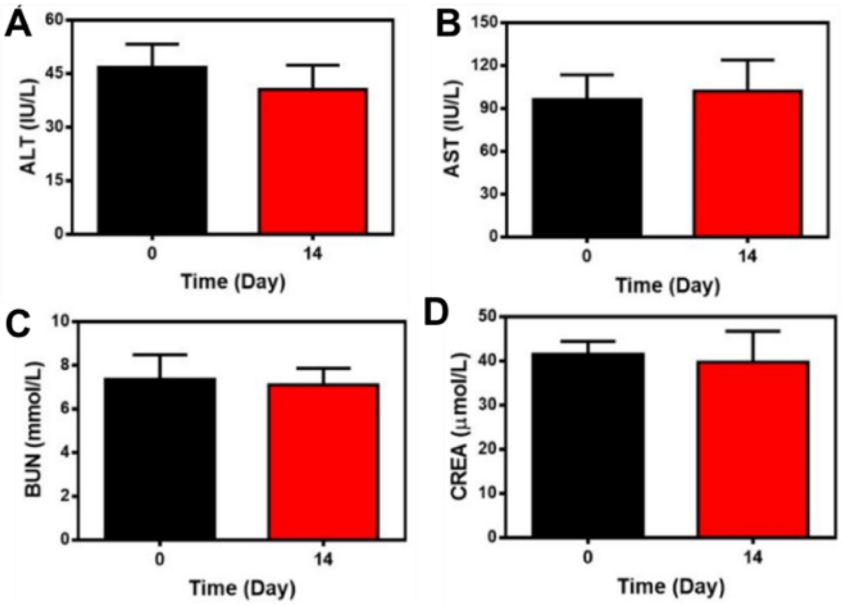

Figure S13 Blood biochemistry results of nude mice before (0 day) and after (14 day) injection of MNS-GOx: ALT (A), AST (B), BUN (C) and CREA (D).

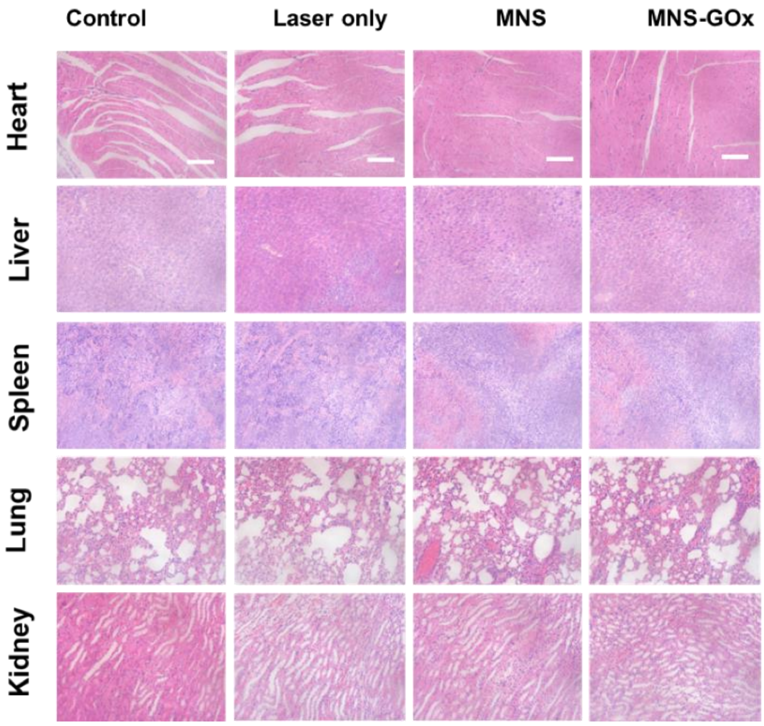

Figure S14 H&E staining images of main organs after 30 d of treatment with different treatments. Scale bar:100 μm.
